# The NLRC4 inflammasome requires IRF8-dependent production of NAIPs

**DOI:** 10.15698/cst2018.06.141

**Published:** 2018-05-22

**Authors:** Ein Lee, Rajendra Karki, Thirumala-Devi Kanneganti

**Affiliations:** 1 Department of Immunology, St. Jude Children’s Research Hospital, Memphis, TN 38105, USA.; 2 Integrated Biomedical Sciences Program, University of Tennessee Health Science Center, Memphis, TN 38163, USA.

**Keywords:** IRF8, NLRC4, Inflammasome, NAIPs, Salmonella

## Abstract

Activation of the NLRC4 inflammasome is crucial for defense against bacterial species that have flagellin or the type III secretion system (T3SS). We have discovered the role of interferon regulatory factor 8 (IRF8) in mediating NLRC4 inflammasome activation. IRF8 is required for the transcription of genes encoding NAIPs, thereby enabling cellular detection of flagellin or T3SS proteins. *In vivo*, IRF8 is important for NLRC4 inflammasome-dependent cytokine production, bacterial clearance, and ultimately, host survival. By introducing IRF8 as a player in inflammasome regulation, our study provides a new perspective on that process.

nflammasomes orchestrate cellular responses to pathogen- or danger-associated molecular patterns by controlling the activation of caspase-1. Inflammasome activation results in enzymatically active caspase-1, which mediates the maturation and release of the pro-inflammatory cytokines IL-1β and IL-18 and induces a form of inflammatory cell death termed pyroptosis. The NLRC4 inflammasome is crucial for protection against infection by bacterial pathogens such as *Salmonella* or *Legionella*. In fact, activators of the NLRC4 inflammasome are strictly limited to pathogenic triggers, because the inflammasome senses bacterial proteins in the cytosol. The NLRC4 inflammasome is unique among inflammasome complexes in that it requires another sensor for ligand recognition. Nucleotide-binding domain and leucine-rich repeat receptor (NLR) family apoptosis inhibitory proteins (NAIPs) are the direct receptors that recognize conserved bacterial proteins. NAIP1 and NAIP2 interact with components of the bacterial type III secretion system (T3SS); specifically, NAIP1 interacts with the needle component of the T3SS and NAIP2 detects the inner rod component. NAIP5 and NAIP6 both serve as sensors for flagellin.

Although there is extensive literature on the regulation of the NLRP3 inflammasome, it has hitherto been unknown how NLRC4 inflammasome activity is controlled. We have demonstrated for the first time that interferon regulatory factor 8 (IRF8) is a critical regulator of NLRC4 inflammasome activation for host defense against Gram-negative bacteria. Previous studies have revealed the contribution of type I interferons and IRFs in bacteria-induced AIM2 and noncanonical NLRP3 inflammasome activation. Thus, our findings also suggest a common cellular function for IRFs in promoting inflammasome activation during bacterial infection.

*Irf8*^−/−^ bone marrow-derived macrophages (BMDMs) infected with *Salmonella* Typhimurium, *Burkholderia thailandensis*, or *Pseudomonas aeruginosa *displayed reduced NLRC4 inflammasome activation when compared to wild-type (WT) BMDMs. The reduced caspase-1 activation in these cells was associated with a significant decrease in IL-18 and IL-1β secretion and in pyroptotic cell death (**Figure 1**). However, the production of inflammasome-independent cytokines such as TNF or IL-6 remained unchanged during these infections. Given that IRF8 is a key transcription factor for macrophage development and function, we looked for differential regulation of innate immune sensors by using microarrays. We found reduced expression of the *Naip2*, *Naip5*, *Naip6*, and *Nlrc4* genes in* S*. Typhimurium-infected *Irf8*^−/−^ BMDMs, as compared to WT BMDMs. We were able to confirm these findings with real-time PCR (RT-PCR), which showed that, compared to WT BMDMs, *Irf8*^−/−^ BMDMs had decreased expression of all the *Naip* genes and *Nlrc4*. Supporting these results, when we re-analyzed a published chromatin immunoprecipitation-sequencing (ChIP-seq) dataset, we found IRF8 binding to the promoter regions of *Naip2*, *Naip5*, and *Naip6* and to the intronic region of *Nlrc4*. Moreover, several biochemical approaches, including targeted ChIP-PCR, luciferase assay, electrophoretic mobility shift assay (EMSA), and reconstitution, verified that IRF8 is a transcriptional activator of *Naip* genes (**Figure 1**).

**Figure 1:**
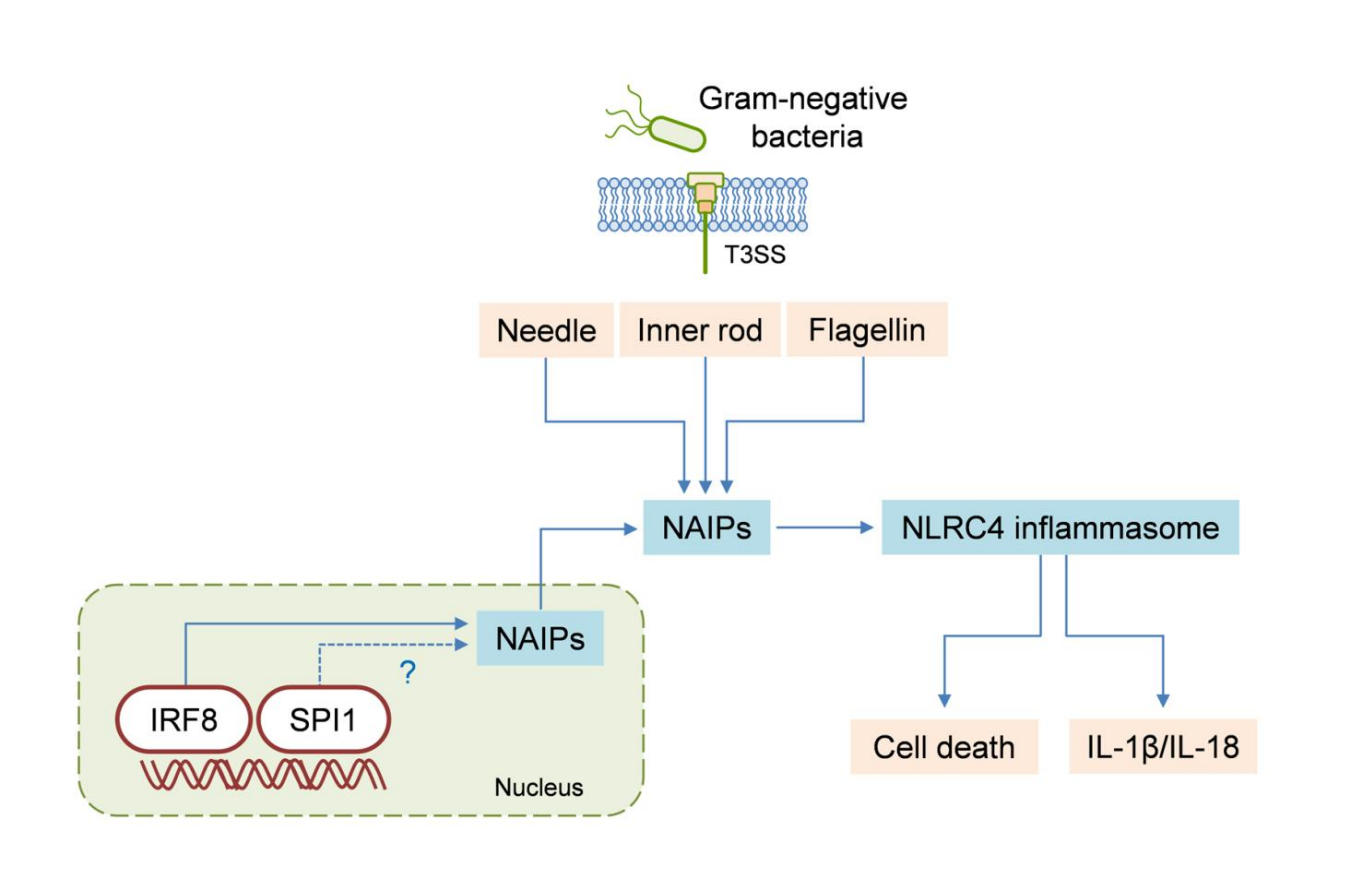
FIGURE 1: IRF8 regulates NLRC4 inflammasome activation. NAIPs are the cytosolic receptors that sense bacterial proteins, specifically the type III secretion system (T3SS) needle, the T3SS inner rod, and flagellin. IRF8 is required for the transcription of genes encoding NAIPs, and SPI1 may also have a role in that process. The detection of bacterial proteins via NAIPs activates the NLRC4 inflammasome, leading to cell death and IL-1β/IL-18 secretion.

The observed defect in NLRC4 inflammasome activation was reproducible *in vivo*; *Irf8*^−/− ^mice were more susceptible than WT mice to infection with *S.* Typhimurium or *B. thailandensis*. *Irf8*^−/− ^mice exhibited accelerated mortality and impaired bacterial clearance when compared to their WT counterparts. The basally lower expression of *Naip* genes in IRF8-deficient mice resulted in defective activation of the NLRC4 inflammasome, which was reflected in the lack of IL-18 production in these mice during bacterial infection. We should note that macrophages are not the only cells to undergo NLRC4 inflammasome activation; there is substantial literature showing that NLRC4 inflammasome activation happens in monocytes, dendritic cells, neutrophils, and lymphocytes. *Irf8*^−/− ^mice infected with *S.* Typhimurium or *B. thailandensis *had reduced expression of *Naip *genes in the spleen and lungs, which contain heterogeneous populations of cells. Thus, inflammasome activation in all these cells may contribute to NLRC4 inflammasome-mediated protection against bacterial pathogens.

Although our study has convincingly demonstrated the role of IRF8 in transcriptional regulation of NAIPs, there are also other mechanisms by which IRF8 could modulate NLRC4 inflammasome activation. One study found that IRF8-dependent autophagy was important for the clearance of *Listeria monocytogenes* in macrophages. As the relation between inflammasomes and autophagy is being increasingly explored, it would be interesting to know how NLRC4 inflammasome activation is affected by this fundamental cellular process. Also, phosphorylation of NLRC4 is required for inflammasome activation, and PKCδ is involved in this event. *Prkcd*, the gene encoding PKCδ, was upregulated after induced expression of IRF8 in a myeloid progenitor cell line. This raises the question as to whether IRF8 could have additional roles upstream of NLRC4 inflammasome activation.

We observed that IRF8 expression in the spleens of *S.* Typhimurium-infected mice was downregulated compared to that in the spleens of uninfected mice. This suggests that a negative-feedback mechanism may restrict overt inflammasome activation during persistent infection. In addition, IRF8-dependent expression of individual *Naip* genes varied among tissues. The expression of *Naip1*, *Naip5*, *Naip6*, and *Nlrc4*, but not of *Naip2*, was basally lower in the spleens of *Irf8*^−/− ^mice than in those of WT mice. However, when we analyzed gene expression in the lungs, we found a significant reduction in *Naip2* expression levels as well. It should be taken into consideration that factors other than IRF8 are also probably involved in the complex regulation of NAIPs* in vivo*. For example, neutrophils do not express IRF8 but nevertheless express NAIPs and can undergo NLRC4 inflammasome activation. In this respect, we suggest that SPI1 (PU.1) regulates the expression of *Naip* genes and the activation of the NLRC4 inflammasome. SPI1 and IRF8 are recruited together to genomic regions containing conserved composite elements. Supporting this idea, our analysis of a published ChIP-seq dataset for SPI1 revealed binding to *Naip1*, *Naip2*, *Naip5*, and *Naip6* loci. We do not know whether SPI1 and IRF8 share binding sites in the promoter regions of *Naip* genes. Going forward, it will be important to assess how IRF8 and SPI1 expression relates to NAIP expression and NLRC4 inflammasome activation in different tissues and cell types.

